# The Diversity of Lipopolysaccharide (O) and Capsular Polysaccharide (K) Antigens of Invasive *Klebsiella pneumoniae* in a Multi-Country Collection

**DOI:** 10.3389/fmicb.2020.01249

**Published:** 2020-06-12

**Authors:** Myeongjin Choi, Nicolas Hegerle, Joseph Nkeze, Shaichi Sen, Sanchita Jamindar, Shamima Nasrin, Sunil Sen, Jasnehta Permala-Booth, James Sinclair, Milagritos D. Tapia, J. Kristie Johnson, Sylla Mamadou, Joshua T. Thaden, Vance G. Fowler, Ana Aguilar, Enrique Terán, Dominique Decre, Florence Morel, Karen Angeliki Krogfelt, Annelie Brauner, Efthymia Protonotariou, Eirini Christaki, Yuichiro Shindo, Yi-Tsung Lin, Andrea L. Kwa, Sadia Shakoor, Ashika Singh-Moodley, Olga Perovic, Jan Jacobs, Octavie Lunguya, Raphael Simon, Alan S. Cross, Sharon M. Tennant

**Affiliations:** ^1^Center for Vaccine Development and Global Health, University of Maryland School of Medicine, Baltimore, MD, United States; ^2^Department of Medicine, University of Maryland School of Medicine, Baltimore, MD, United States; ^3^Department of Pediatrics, University of Maryland School of Medicine, Baltimore, MD, United States; ^4^Department of Pathology, University of Maryland School of Medicine, Baltimore, MD, United States; ^5^Centre pour le Développement des Vaccins, Bamako, Mali; ^6^Division of Infectious Diseases, Duke University Medical Center, Durham, NC, United States; ^7^Department of Medicine, Division of Infectious Diseases and International Health, Duke University School of Medicine, Durham, NC, United States; ^8^Duke Clinical Research Institute, Durham, NC, United States; ^9^Colegio de Ciencias de la Salud e Instituto de Microbiologia, Universidad San Francisco de Quito, Quito, Ecuador; ^10^Département de Bactériologie, Centre d’Immunologie et des Maladies Infectieuses-Paris, Cimi-Paris, INSERM U1135, AP-HP, Sorbonne Université, Hôpitaux Universitaires Est Parisien, Paris, France; ^11^Statens Serum Institut, Copenhagen, Denmark; ^12^Department of Microbiology, Tumor and Cell Biology, Division of Clinical Microbiology, Karolinska Institutet, Karolinska University Hospital, Stockholm, Sweden; ^13^Department of Microbiology, AHEPA University Hospital, Thessaloniki, Greece; ^14^Department of Medicine, AHEPA University Hospital, Thessaloniki, Greece; ^15^Medical School, University of Cyprus, Nicosia, Cyprus; ^16^Department of Respiratory Medicine, Graduate School of Medicine, Nagoya University, Nagoya, Japan; ^17^Division of Infectious Diseases, Department of Medicine, Taipei Veterans General Hospital, Taipei, Taiwan; ^18^Institute of Emergency and Critical Care Medicine, National Yang-Ming University, Taipei, Taiwan; ^19^Department of Pharmacy, Singapore General Hospital, Singapore, Singapore; ^20^Emerging Infectious Diseases, Duke-National University of Singapore Medical School, Singapore, Singapore; ^21^Department of Pharmacy, Faculty of Science, National University of Singapore, Singapore, Singapore; ^22^Departments of Pathology and Pediatrics, Aga Khan University, Karachi, Pakistan; ^23^National Institute for Communicable Diseases, A Division of the National Health Laboratory Service, School of Pathology, Faculty of Health Sciences, University of the Witwatersrand, Johannesburg, Johannesburg, South Africa; ^24^Department of Clinical Sciences, Institute of Tropical Medicine Antwerp, Antwerp, Belgium; ^25^Department of Microbiology, Immunology and Transplantation, KU Leuven, Leuven, Belgium; ^26^Department of Clinical Microbiology and Microbiology, National Institute for Biomedical Research, University Hospital of Kinshasa, Kinshasa, Democratic Republic of Congo

**Keywords:** *Klebsiella pneumoniae*, multidrug resistance, vaccine, O antigen, K antigen

## Abstract

*Klebsiella pneumoniae* is a common cause of sepsis and is particularly associated with healthcare-associated infections. New strategies are needed to prevent or treat infections due to the emergence of multi-drug resistant *K. pneumoniae*. The goal of this study was to determine the diversity and distribution of O (lipopolysaccharide) and K (capsular polysaccharide) antigens on a large (>500) global collection of *K. pneumoniae* strains isolated from blood to inform vaccine development efforts. A total of 645 *K. pneumoniae* isolates were collected from the blood of patients in 13 countries during 2005–2017. Antibiotic susceptibility was determined using the Kirby-Bauer disk diffusion method. O antigen types including the presence of modified O galactan types were determined by PCR. K types were determined by multiplex PCR and *wzi* capsular typing. Sequence types of isolates were determined by multilocus sequence typing (MLST) targeting seven housekeeping genes. Among 591 isolates tested for antimicrobial resistance, we observed that 19.3% of isolates were non-susceptible to carbapenems and 62.1% of isolates were multidrug resistant (from as low as 16% in Sweden to 94% in Pakistan). Among 645 isolates, four serotypes, O1, O2, O3, and O5, accounted for 90.1% of *K. pneumoniae* strains. Serotype O1 was associated with multidrug resistance. Fifty percent of 199 tested O1 and O2 strains were *gmlABC*-positive, indicating the presence of the modified polysaccharide subunit D-galactan III. The most common K type was K2 by both multiplex PCR and *wzi* capsular typing. Of 39 strains tested by MLST, 36 strains were assigned to 26 known sequence types of which ST14, ST25, and ST258 were the most common. Given the limited number of O antigen types, diverse K antigen types and the high multidrug resistance, we believe that an O antigen-based vaccine would offer an excellent prophylactic strategy to prevent *K. pneumoniae* invasive infection.

## Introduction

*Klebsiella pneumoniae* is a common Gram-negative bacterial cause of sepsis that contributes to high mortality and morbidity in hospitalized, immunosuppressed, and chronically ill patients. *Klebsiella* spp. were the third most common cause of healthcare-associated infections (9.9%) in acute-care hospitals in United States in 2011 after *Clostridioides difficile* (12.1%) and *Staphylococcus aureus* (10.7%) (Magill et al., 2014). A follow-up study was performed in 2015 and showed that the prevalence of healthcare-associated infections was decreased compared to 2011. Further, *Klebsiella* spp. were responsible for ∼5% of these infections with *C. difficile* (15%), *S. aureus* (11%) and *Escherichia coli* (10%) being the most common (Magill et al., 2018). Similarly, *K. pneumoniae* was responsible for 12% of healthcare-associated pneumonia infections in a point prevalence survey in acute care hospitals in the European Union in 2011–2012 (Walter et al., 2018). Many additional studies have highlighted the importance of *K. pneumoniae* as a cause of healthcare-associated infections (Meatherall et al., 2009; Folgori et al., 2016; Cai et al., 2017; Baykara et al., 2018).

The occurrence of multidrug-resistant (MDR) *K. pneumoniae* has been considered as an urgent threat worldwide (Lee et al., 2017). Limited therapeutic options for infection with MDR *K. pneumoniae* results in increased mortality, longer hospital stays and inflated healthcare costs (Cerceo et al., 2016). Given the emergence of multidrug resistant isolates, prevention of *K. pneumoniae* infections has become of paramount importance. Several groups have developed vaccines to prevent *K. pneumoniae* infection and these target capsular polysaccharide, extracellular vesicles and outer membrane proteins (Cryz et al., 1986a; Edelman et al., 1994; Chhibber et al., 2005; Lundberg et al., 2013; Lee et al., 2015). Likewise, we are developing a quadrivalent conjugate vaccine that targets the O1, O2, O3, and O5 polysaccharides of *K. pneumoniae* (Hegerle et al., 2018). We and others have shown that antibodies directed against the O polysaccharide are protective against wild-type *K. pneumoniae* in lethal infection models (Clements et al., 2008; Hegerle et al., 2018).

*Klebsiella pneumoniae* capsular polysaccharide (K antigen) and lipopolysaccharide (O antigen) are important virulence factors that can activate the innate immune system (Simoons-Smit et al., 1986; Shankar-Sinha et al., 2004). Additionally, these two antigens are used to differentiate *K. pneumoniae* isolates. More than 77 K antigens have been defined to date (Follador et al., 2016). Trautmann et al. (1997) determined that over 80% of *K. pneumoniae* isolates from Denmark belong to O1, O2, O3, and O5. This finding is corroborated by Follador et al. (2016) who sequenced more than 500 human and environmental isolates from United States, United Kingdom, Australia, Singapore, Indonesia, Laos, Nepal and Vietnam. Of these, only 134 out of the 500 isolates were from humans; 47 strains were from urine, 67 strains were isolated from blood, and 20 strains were from sputum (Follador et al., 2016). Nine serotypes have been proposed but in different combinations by separate groups O1, O2ab (O2a), O2a/O9 (O2aeh), O3, O4, O5, O7, O11, and O12 was proposed by Trautmann et al. (1997) whereas O1, O2, O2ac, O3, O4, O5, O7, O8, and O12 was proposed by Hansen et al. (1999). The O8 polysaccharide is similar to the O1 antigen and O9 is a subtype of the O2 antigen (Ørskov and Ørskov, 1984; Kelly et al., 1993). Serotyping O2 isolates is particularly complex due to the various O2 subtypes (O2a, O2ac, O2aeh, and O2afg) (Clarke et al., 2018). LPS O antigens are composed of D-galactans in O1 and O2 and mannans in O3 and O5 strains. Among galactans, D-galactan I was found in O1 (with D-galactan II), O2a, and O2ac isolates (with 2c polysaccharide) (Whitfield et al., 1991, 1992). Recently, D-gal III has been identified as a component of the LPS of O1 and O2 strains; D-gal III is a product of conversion of the D-gal I disaccharide by the *gmlABC* operon (Stojkovic et al., 2017). More recently, multilocus sequence typing (MLST) has replaced O and K typing as the most common method used to type *K. pneumoniae* isolates (Diancourt et al., 2005).

The goal of this study was to determine the global diversity and distribution of O and K antigens on a large (>500) global collection of *K. pneumoniae* strains isolated from blood. This data will help to determine whether a glycoconjugate vaccine that targets a limited number of O antigens would provide coverage against the majority of *K. pneumoniae* bacteremic strains that are currently circulating worldwide. We are particularly interested in evaluating prevalence amongst blood culture isolates as our intended primary endpoint for a Phase 3 clinical trial would be prevention of systemic infections.

## Materials and Methods

### Bacterial Strains

All *K. pneumoniae* isolates were collected from the blood of patients in 13 countries during 2005–2017 (Table 1). All isolates were identified using standard microbiological procedures. A random sample of *K. pneumoniae* strains collected from the Americas (United States and Ecuador), Europe (France, Denmark, and Sweden), Asia (Japan, Taiwan, Singapore, and Pakistan) and Africa (South Africa, DR Congo, and Mali), irrespective of antimicrobial resistance, was selected for analysis. However, isolates from Greece were inadvertently selected from a multi drug resistance collection. The number of isolates collected each year is shown for each country in Supplementary Figure S1. Altogether, 645 *K. pneumoniae* isolates were shipped to the Center for Vaccine Development and Global Health at the University of Maryland School of Medicine for subsequent testing and analysis. *K. pneumoniae* B5055 (O1), 7380 (O2), 390 (O3), Mich.61 (O4), 4425/51 (O5), 889 (O8), 1205 (O9), and 708 (O12) were used as reference strains for the Ørskov and Ørskov O typing scheme (Ørskov and Ørskov, 1984; Trautmann et al., 2004). *K. pneumoniae* strains were grown in animal product-free HY-Soy (HS) media [0.5% sodium chloride, 1% Hy-Soy (TEKnova, Hollister, CA, United States), 0.5% Hy-Yest 444 (Kerry Biosciences, Beloit, WI, United States) at 37°C. Genomic DNA was extracted using the GenElute Bacterial Genomic DNA kit (Sigma-Aldrich, St. Louis, MO, United States) following the manufacturer’s instructions. DNA was extracted from 1.5 ml of overnight bacterial culture and eluted into 200 μl of elution solution (10 mM Tris–HCl, 0.5 mM EDTA, pH 9.0) and 2 μl of DNA was used for polymerase chain reaction (PCR).

**TABLE 1:** Clinical *Klebsiella pneumoniae* isolates used in this study.

Region	Country	Institute	Years	Number of isolates
Americas	United States	University of Maryland Medical Center	2010–2015	107
	United States	Duke University Medical Center	2008–2015	73
	Ecuador	Instituto Nacional de Investigación en Salud Publica Universidad San Francisco de Quito	2016	13
Europe	France	Saint-Antoine Hospital	2008–2011	11
	Denmark	Statens Serum Institute	2017	34
	Sweden	Karolinska University Hospital, Stockholm	2015	25
	Greece	University General Hospital of Thessaloniki (AHEPA)	2014–2016	54
Asia	Japan	Nagoya University Graduate School of Medicine	2010–2015	19
	Taiwan	Taipei Veterans General Hospital	2012–2013	6
	Singapore	Singapore General Hospital	2012–2016	25
	Pakistan	Aga Khan University	2015–2017	71
Africa	South Africa	National Institute for Communicable Diseases	2011–2012	96
	DR Congo	University Hospital of Kinshasa	2015	83
	Mali	Center for Vaccine Development-Mali	2005–2012	28

### Antimicrobial Susceptibility Test

Antimicrobial susceptibility was determined by the Kirby-Bauer disk diffusion method. The following antibiotics were tested in this study: amikacin, ampicillin/sulbactam, aztreonam, cefazolin, cefepime, ceftriaxone, ertapenem, gentamicin, levofloxacin, meropenem, piperacillin-tazobactam, and tetracycline. Antimicrobial impregnated disks (BD BBL^TM^ Sensi-Disk^TM^) were purchased from Becton, Dickinson and Company (Franklin Lakes, NJ, United States). Antimicrobial susceptibility was determined by measuring the diameter of the zone of inhibition and the results were interpreted according to Clinical and Laboratory Standards Institute (CLSI) guidelines (M100-S29). MDR *K. pneumoniae* was defined as non-susceptible to at least one agent in three or more antibiotic classes. Non-MDR was defined as non-susceptible to less than three antimicrobial categories or fully susceptible (Magiorakos et al., 2012). Isolates from Greece were part of an MDR collection and were thus excluded from all analyses involving antimicrobial resistance to avoid bias from non-randomly selected strains.

### Typing O Antigens and Genetic Analysis of D-Galactan Types

O typing was performed by PCR, using a method described by Fang et al. (2016) except that we did not differentiate O2ac from O2a. O1, O2, O3, O4, O5, O8, O9, and O12 were determined (identification of O7 which is rare was not done). Briefly, genomic DNA (>10 ng) was used as the template, Dreamtaq Green PCR Master Mix (Thermo Fisher Scientific, Waltham, MA, United States) was used for PCR, with 0.4 μM primers and 2 mM Mg^2+^ in a final reaction volume of 50 μl. The following cycling conditions were used: 95°C for 10 min, followed by 30 cycles of 95°C for 30 s, 60°C for 30 s, and 72°C for 1 min, then a final extension at 72°C for 10 min. DNA fragment size was determined on a 1% agarose gel. D-galactan III-possessing strains were identified by screening O1 and O2 strains randomly selected from different regions for the *gmlABC* locus by PCR (Szijártó et al., 2016). The geographic distribution of O types was visualized using an online tool^1^.

### K Typing

Two methods were used to K type *K. pneumoniae*: multiplex PCR and *wzi* gene sequencing. K1, K2, K5, K20, K54, and K57 were identified by multiplex PCR described by Turton et al. (2010). Briefly, multiplex PCR was performed using the Qiagen Multiplex PCR kit (Qiagen, Hilden, Germany) with 0.2 μM primers and 3 mM Mg^2+^ in a final reaction volume of 25 μl. Cycling parameters were 95°C for 15 min, followed by 35 cycles of 94°C for 30 s, 58°C for 90 s and 72°C for 90 s, and a final extension at 72°C for 10 min. Amplicons were separated on a 1.5% agarose gel.

The *wzi* gene was sequenced and K types determined as described by Brisse et al. (2013). PCR amplification of *wzi* was achieved by using PCR buffer containing 2 mM dNTP’s, 0.4 μM primers, and 1 U of Green Taq DNA polymerase (Genscript, Piscataway, NJ, United States). Cycling conditions were as follows: 95°C for 3 min, followed by 40 cycles of 95°C for 30 s, 56°C for 45 s and 72°C for 40 s, and a final extension at 72°C for 5 min. After DNA purification using a QIAquick PCR Purification kit (Qiagen), amplicons were sequenced (Genewiz, South Plainfield, NJ, United States) and chromatograms were reviewed and sequences trimmed using Geneious software (Biomatters Ltd., Auckland, New Zealand). The *wzi* alleles and K types were identified from the *K. pneumoniae* sequence typing database at http://bigsdb.web.pasteur.fr. Phylogenetic trees of *wzi* alleles were constructed using MEGA version 7.0 based on the neighbor-joining method (100 bootstrap replicates) and Jukes-Cantor distance (Brisse et al., 2013; Martin et al., 2016).

### Multilocus Sequence Typing (MLST) Analysis

Multilocus sequence typing with seven genes (*gapA*, *infB*, *mdh*, *pgi*, *phoE*, *rpoB*, and *tonB*) was performed on strains according to the protocol described on the *K. pneumoniae* MLST website^2^ (Diancourt et al., 2005). The MLST database was used to assign alleles and sequence types (STs). Clonal complexes (CCs) were defined as groups for which MLST profiles showed only one allelic mismatch with at least one other member of the group (Bialek-Davenet et al., 2014).

### Statistical Analysis

The proportion of susceptible versus resistant strains for each O type was analyzed by Chi-square analysis with Yate’s correction using GraphPad Prism version 6.05 software (GraphPad Software, San Diego, United States). The significance level was *p* < 0.05.

## Results

### Antimicrobial Resistance

All 54 MDR isolates from Greece were resistant to ampicillin-sulbactam, cefazolin, cefepime, ceftriaxone, ertapenem, meropenem, and piperacillin-tazobactam (data not shown). These isolates were excluded from subsequent analyses (Supplementary Figure S2). High resistance rates to ampicillin-sulbactam (60.9%) and cefazolin (77.3%) were observed among 591 global isolates (see Supplementary Table S2). Resistance to carbapenems, which are used as a last resort for serious multidrug resistant infections (Meletis, 2016), was observed to be 19.3% (Table 2). The highest resistance rate against carbapenems was observed in Asia (51.2% of Asian isolates were resistant).

**TABLE 2:** Antibiotic resistance of 591 *Klebsiella pneumoniae* isolated from blood.

Region	Country^b^	Number of isolates	No. of isolates (%) non-susceptible to^a^								MDR^c^
			
			Aminogly- cosides	Penicillins + β-lactamase inhibitors	Non-extended spectrum cephalosporins	Extended spectrum cephalosporins	Carbapenems	Monobactams	Fluoro- quinolones	Tetracyclines	
Global		591	266 (45.0)	382 (64.6)	457 (77.3)	338 (57.2)	114 (19.3)	273 (46.2)	243 (41.1)	248 (42.0)	367 (62.1)
Americas	United States^d^	107	29 (27.1)	59 (55.1)	80 (74.8)	52 (48.6)	20 (18.7)	22 (20.6)	35 (32.7)	27 (25.2)	57 (53.3)
	United States^e^	73	10 (13.7)	32 (43.8)	51 (69.9)	23 (31.5)	9 (12.3)	4 (5.5)	17 (23.3)	19 (26.0)	28 (38.4)
	Ecuador	13	9 (69.2)	10 (76.9)	13 (100)	9 (69.2)	5 (38.5)	9 (69.2)	9 (69.2)	9 (69.2)	9 (69.2)
	Total	193	48 (24.9)	101 (52.3)	144 (74.6)	84 (45.5)	34 (17.6)	35 (18.1)	61 (31.6)	55 (28.5)	94 (48.7)
Europe	France	11	1 (9.1)	6 (54.5)	3 (27.3)	3 (27.3)	0 (0)	3 (27.3)	1 (9.1)	5 (45.5)	3 (27.3)
	Denmark	34	1 (2.9)	13 (38.2)	23 (67.6)	7 (20.6)	3 (8.8)	3 (8.8)	6 (17.6)	7 (20.6)	11 (32.4)
	Sweden	25	1 (4)	7 (28)	8 (32)	1 (4)	1 (4)	1 (4)	3 (12)	3 (12)	4 (16)
	Total	70	3 (4.3)	26 (37.1)	34 (48.6)	11 (15.7)	4 (5.7)	7 (10)	10 (14.3)	15 (21.4)	18 (25.7)
Asia	Japan	19	1 (5.3)	8 (42.1)	9 (47.4)	4 (21.1)	0 (0)	3 (15.8)	4 (21.1)	6 (31.6)	6 (31.6)
	Taiwan	6	1 (16.7)	2 (33.3)	3 (50)	0 (0)	2 (33.3)	0 (0)	1 (16.7)	2 (33.3)	1 (16.7)
	Singapore	25	10 (40.0)	16 (64.0)	21 (84.0)	11 (44.0)	7 (28.0)	8 (32.0)	12 (48.0)	10 (40.0)	14 (56.0)
	Pakistan	71	54 (76.1)	65 (91.5)	69 (97.2)	64 (90.1)	53 (74.6)	61 (85.9)	65 (91.5)	41 (57.7)	67 (94.4)
	Total	121	66 (54.5)	91 (75.2)	102 (84.3)	79 (65.3)	62 (51.2)	72 (59.5)	82 (67.8)	59 (48.8)	88 (72.7)
Africa	South Africa	96	67 (69.8)	80 (83.3)	86 (89.6)	76 (79.2)	8 (8.3)	73 (76)	50 (52.1)	41 (42.7)	77 (80.2)
	DR Congo	83	63 (75.9)	64 (77.1)	70 (84.3)	68 (81.9)	5 (6.0)	68 (81.9)	31 (37.3)	66 (79.5)	69 (83.1)
	Mali	28	19 (67.9)	20 (71.4)	21 (75.0)	20 (71.4)	1 (3.6)	18 (64.3)	9 (32.1)	12 (42.9)	21 (75.0)
	Total	207	149 (72.0)	164 (79.2)	177 (85.5)	164 (79.2)	14 (6.8)	159 (76.8)	90 (43.5)	119 (57.5)	167 (80.7)

*^*a*^Amikacin and gentamicin (aminoglycosides); piperacillin/tazobactam and ampicillin/sulbactam (penicillins + ß-lactamase inhibitors); cefazolin (non-extended spectrum cephalosporins, the 1st and 2nd generations); ceftriaxone and cefepime (extended spectrum cephalosporins, the 3rd and 4th generations); meropenem and ertapenem (carbapenems); aztreonam (monobactams); levofloxacin (fluoroquinolones); tetracycline (tetracycline) were used in this study. ^*b*^Isolates collected from Greece were not included. ^*c*^MDR defined as resistant to at least three antibiotic classes. ^*d*^Isolates were obtained from University of Maryland Medical Center. ^*e*^Isolates were obtained from Duke University Medical Center.*

Overall, we observed 62.1% MDR globally (Table 2). Multidrug resistance varied by region with higher proportions observed in Africa (80.7%) and Asia (72.7%) than in the Americas (48.7%) or Europe (25.7%). In the United States, 53.3 and 38.4% of isolates collected from University of Maryland Medical Center and Duke University Medical Center were multidrug-resistant, respectively. MDR *K. pneumoniae* isolates were observed across Europe with the percentage ranging from 16 to 32.4%. The percentage of MDR isolates collected from Japan (31.6%) and Taiwan (16.7%) were lower than that of Singapore (56%) and Pakistan (94.4%).

### O Types and D-Galactan Types

**FIGURE 1:**
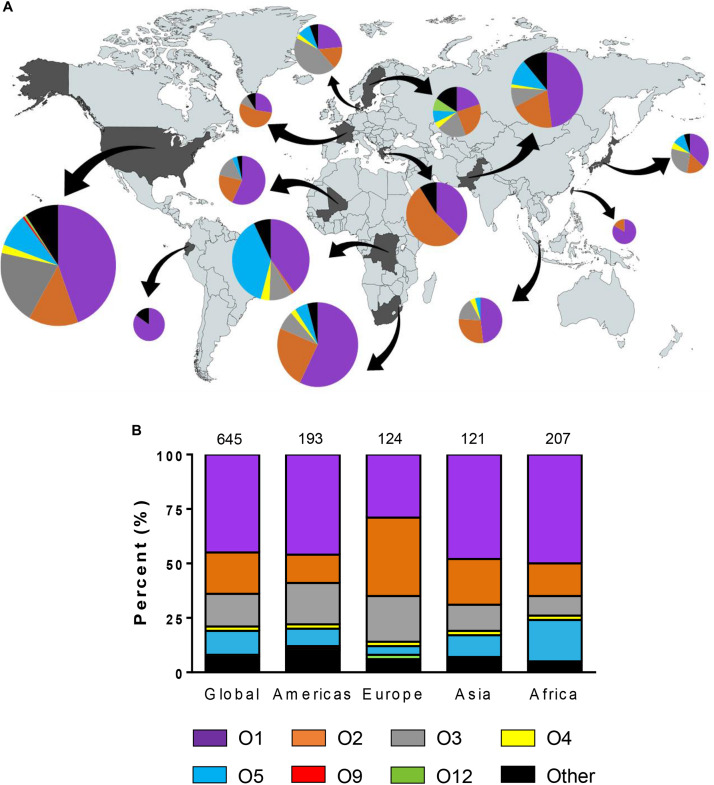
Geographic distribution of *Klebsiella pneumoniae* O types. **(A)** Diversity of O types by country. The size of each pie chart represents the number of isolates tested from each country. Arrows indicate each country from which strains were isolated. **(B)** Diversity of O types by region. The numbers on the top of the graph represent the number of isolates belonging to each region. No O8 strains were found in this study.

Among 645 *K. pneumoniae* isolates, 44.8% were O1, 19.5% were O2, 14.9% were O3, and 10.9% were O5. Collectively, these four O types accounted for 90.1% of *K. pneumoniae* strains in 13 countries (Figure 1). One O9 isolate in United States and 3 O12 isolates in two countries (United States and Sweden) were found while 14 O4 isolates were found globally. No O8 isolates were identified. Although we observed diverse profiles of O types between countries, the total percent of O1, O2, O3, and O5 isolates collectively ranged from 72% in Sweden to as high as 100% in Greece. We also evaluated the serotype distribution in the United States over time and found that the prevalence of O1, O2, O3, and O5 remained fairly stable between 2011 and 2015 (Supplementary Figure S3). We observed an increase in the percentage of O1 in the United States from 44% in 2011 to 50% in 2015 and in O5 from 2.2% in 2011 to 10.7% in 2015. In contrast, serotype O2 decreased from 20 to 3.6% though statistically significant differences were not reached due to the small numbers. Some *K. pneumoniae* O1 and O2 strains have been shown to possess a modified galactan O-antigen type (Stojkovic et al., 2017). We screened 199 O1 and O2 isolates for the presence of *gmlABC* which encodes this D-galactan III antigen. We detected *gmlABC* in 49.7% of isolates tested. Our data suggests that D-galactan I is more common in Europe than in other regions (Table 3).

**TABLE 3:** Distribution of D-galactan I (*gmlABC*-negative) and III (*gmlABC*-positive) in O1 and O2 *Klebsiella pneumoniae* in 13 countries.

Region	No. of *gmlABC*-negative strains (%)^a^	No. of *gmlABC*-positive strains (%)^b^	ND^c^	Total
Americas	24 (48%)	26 (52%)	0 (0%)	50 (100%)
Europe	31 (62%)	19 (38%)	0 (0%)	50 (100%)
Asia	20 (40.8%)	27 (55.1%)	2 (4.1%)	49 (100%)
Africa	20 (40%)	27 (54%)	3 (6%)	50 (100%)
Total	95 (47.8%)	99 (49.7%)	5 (2.5%)	199 (100%)

*^*a*^As determined by amplification of a 2.1-kb PCR product. ^*b*^As determined by amplification of a 5-kb PCR product. ^*c*^ND, not determined (no amplification).*

**FIGURE 2:**
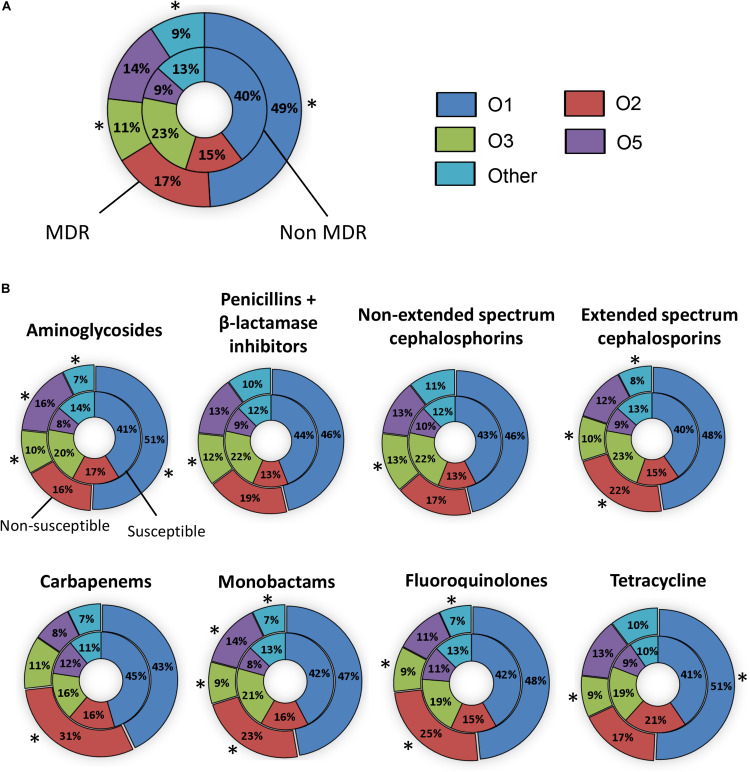
The prevalence of O types in invasive *Klebsiella pneumoniae* that are susceptible or resistant to antibiotics. **(A)** The percentage of O types amongst non-MDR (inner pie chart) and MDR isolates (outer pie chart). **(B)** The percentage of O types amongst isolates susceptible (inner pie chart) and non-susceptible (outer pie chart) to each antibiotic. Statistically significant differences (*P* ≤ 0.05) between susceptible and non-susceptible isolates are indicated by asterisks (^∗^). Isolates collected from Greece were not included.

To determine if particular serotypes were associated with antibiotic resistance, we evaluated the serotype distribution amongst susceptible and non-susceptible strains for each antibiotic class and for MDR versus non-MDR strains. The serotype distribution observed among 367 MDR isolates was 49% for O1, 17% for O2, 11% for O3, and 14% for O5, respectively (Figure 2A). There were significantly more O1 and fewer O3 strains in the MDR group compared to the non-MDR group (*p* < 0.05). The percentage of O serotypes in the susceptible and non-susceptible groups to each antibiotic is shown in Figure 2B. There were significantly more O1 strains that were non-susceptible to aminoglycosides and tetracycline compared to susceptible (*p* < 0.05). Likewise, there were more O2 strains that were not susceptible to extended spectrum cephalosporins, carbapenems, monobactams, and fluoroquinolones than susceptible. There were more O5 strains that were non-susceptible to aminoglycosides and monobactams than susceptible. In contrast, O3 strains were more likely to be susceptible than non-susceptible (to all classes except carbapenems).

### K Types and Phylogenetic Diversity of *wzi* Sequences

**FIGURE 3:**
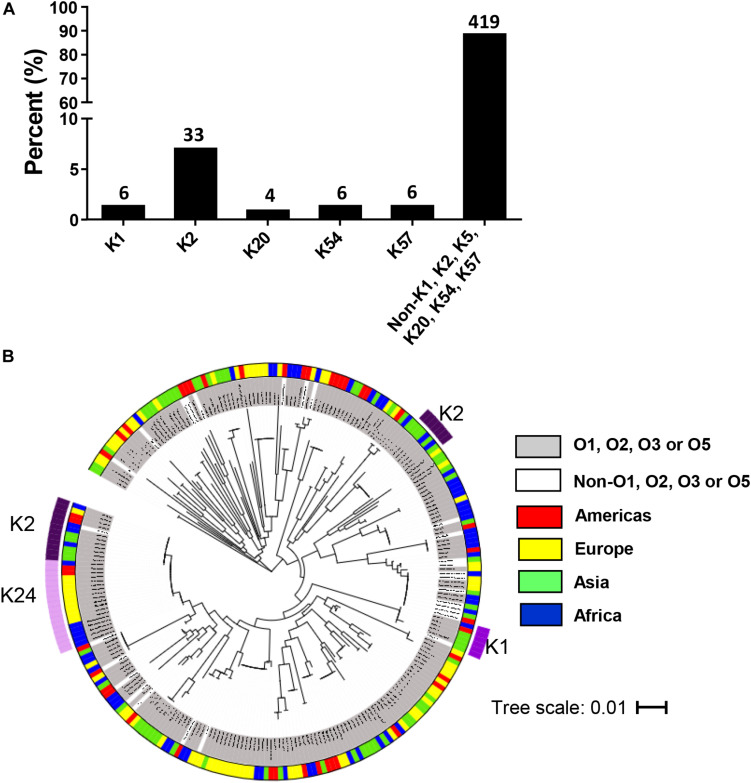
Distribution of K types in invasive *Klebsiella pneumoniae* isolates in the multi-country collection. **(A)** K types by multiplex PCR among 474 isolates. The numbers on top of the bars represent the number of isolates belonging to each K type. **(B)** Phylogenetic tree for *wzi* sequence of 265 invasive randomly selected strains. The scale bar represents the amount of genetic change; 0.01 equals 1 change per 100 nucleotide sites. The categories of O serotypes and the geographical origins are represented by colors as indicated.

The most common K type by multiplex PCR among 474 strains was K2 (7%). However, 88.4% of isolates were negative for K1, K2, K5, K20, K54, and K57 and therefore could not be typed using this method (Figure 3A). Of 519 isolates, 451 possessed known *wzi* alleles (149 *wzi* alleles), of which 298 isolates had known K types (47 K types). Eight new alleles were deposited into public repositories^3^. The most common K types were K2 (6%) and K24 (7%) (Supplementary Figure S4). A phylogenetic tree of *wzi* types from 265 randomly selected isolates is shown in Figure 3B. 89.4% of the strains included in this analysis were O1, O2, O3, and O5 and possessed diverse *wzi* sequences. K2 was identified in two clades according to different *wzi* sequences, *wzi*72 and *wzi*2.

### Sequence Types

Sequence types were determined among 10 randomly selected O1:K2 isolates to assess whether *K. pneumoniae* O1:K2, which are highly pathogenic, share common ancestry lineages worldwide (Yeh et al., 2016). The most common ST among 10 O1:K2 serotype isolates was ST14 (50%, 5 isolates), followed by ST25 (40%, 4 isolates) and ST86 (10%, 1 isolate) (Table 4). All O1:K2 serotype isolates collected from America and Asia were ST14 and ST25. Three different STs were found in four O1:K2 serotype isolates collected from Africa.

**TABLE 4:** Distribution of sequence types in O1:K2 *Klebsiella pneumoniae* isolates.

Region	Isolate	Year	ST
Americas	12-00541	2012	14
	15-02188	2015	14
	15-00479	2015	14
Asia	NUH5204	2015	25
	NUH5279	2015	25
	EC1911	2016	14
Africa	7032	2012	25
	7029	2012	14
	4630327	2012	86
	4897/4	2015	25

*ST, sequence type.*

To determine if particular STs are associated with a particular serotype from a single geographic location, 29 randomly selected O1, O2, O3, and O5 isolates from University of Maryland Medical Center were tested by MLST and 22 known STs were identified (see Supplementary Table S1). Of the 29 strains, 9 were O1 (31%), 6 were O2 (21%), 8 were O3 (28%), 2 were O4 (7%) and 4 were O5 (14%). While CC14 (2 ST15 isolates) and CC258 (3 ST258 and 1 ST1458) were found, another seven ST singletons were also identified among O1 and O2 isolates. All O3 and O5 isolates were singletons. Two new STs (ST2589 and ST2590) in O1 isolates and one ST2591 in O5 isolates were identified, respectively. We observed multiple STs for each O type.

## Discussion

The objective of this study was to determine whether a vaccine that targets *K. pneumoniae* O1, O2, O3, and O5 antigens would provide coverage against the majority of bacteremic strains circulating worldwide. In this study, we examined rates of antibiotic resistance, the distribution and diversity of O antigens, K antigens, and STs and among 645 invasive clinical isolates collected from 13 countries. This is the largest study of invasive *K. pneumoniae* that has been performed to date with isolates coming from diverse geographic locations.

Carbapenems have been considered as a treatment of last choice for multidrug-resistant *K. pneumoniae* (Meletis, 2016; Pennini et al., 2017). Recently, the increased emergence of *K. pneumoniae* resistant to those antibiotics has become a serious problem worldwide. We observed the high rate of non-susceptibility to carbapenems (Table 2), suggesting that antibiotic treatment options for multidrug-resistant *K. pneumoniae* are becoming limited. We observed higher multidrug resistance (62.1%) than Hackel et al. (2016) who found 30.1% MDR out of 9,098 *K. pneumoniae* isolates collected in 2012–2014 from 176 geographic sites. These strains were isolated predominantly from intra-abdominal infections, urinary tract infections, skin and soft tissue infections and lower respiratory tract infections. Unlike our collection, only 3.8% of the isolates were from blood. Further, the higher proportion of multidrug resistant organisms that we observed in our study may be due to inclusion of isolates from sub-Saharan Africa and South Asia which show high rates of MDR (Investigators of the Delhi Neonatal Infection Study (DeNIS) collaboration, 2016; Henson et al., 2017; Musicha et al., 2017). Hackel et al. described isolates from South Africa but no other African countries and no isolates from India, Bangladesh or Pakistan (Hackel et al., 2016).

We found that 90.1% of the *K. pneumoniae* isolates were O1, O2, O3, or O5, consistent with previous reports (Figure 1; Trautmann et al., 1997; Follador et al., 2016). O1 and O2 *K. pneumoniae* isolates have been considered important pathogens due to their high prevalence, virulence and rate of antibiotic resistance (Fang et al., 2016; Szijártó et al., 2016; Pennini et al., 2017). While D-galactan I antigen was first identified as a repeat unit of O polysaccharide in O1 and O2 isolates, some O1 and O2 strains were recently reported to possess a modified D-galactan I, called D-galactan III (Whitfield et al., 1992; Szijártó et al., 2016; Stojkovic et al., 2017). In this study, 49.7% isolates carried the *gmlABC* operon which is associated with D-gal III synthesis. Similarly, Szijártó et al. (2016) reported that 40% of O1 isolates and 68% of O2 isolates possess the *gmlABC* operon (Szijártó et al., 2016). These results imply that an O-polysaccharide-based vaccine would need to ensure that antibodies recognize both the D-gal III epitope as well as the D-gal I epitope to achieve maximal coverage (Stojkovic et al., 2017).

Although O1 and O2 strains are the most common serotypes globally (64.3%), 25.7% of strains tested were determined to be O3 or O5. These findings suggest that vaccines for preventing infection caused by O3 and O5 *K. pneumoniae* are also necessary. No O8 isolate was found among 645 *K. pneumoniae* in line with that seen in studies by other investigators (Follador et al., 2016). It should be noted that *K. pneumoniae* O1 and O8 O polysaccharide are serologically similar and O1 OPS would likely protect against O8 strains (Trautmann et al., 1997). O12 isolates have been isolated from several locations in Europe including Spain, Denmark, and United Kingdom (Hansen et al., 1999; Follador et al., 2016). We found only 3 O12 isolates from United States and Sweden. Thus, targeting O1, O2, O3, and O5, but not O12, would be important for development of a broad spectrum vaccine against *K. pneumoniae*. In the current study, the most common O type was O1, regardless of antibiotic resistance, which is associated with high bacterial dissemination and colonization into organs (Hsieh et al., 2014). Pennini et al. (2017) reported that O2 was the dominant serotype of MDR *K. pneumoniae* and associated with ß-lactam and carbapenem-resistance (Pennini et al., 2017). In our study, although O2 was associated with resistance to extended spectrum cephalosporins, carbapenems, monobactams, and fluoroquinolones (Figure 2B), O1 was the dominant serotype of MDR *K. pneumoniae* and associated with resistance to aminoglycosides and tetracycline. O5 strains were associated with non-susceptibility to aminoglycosides, and monobactams. In the Pennini et al. (2017) study, 57.7% of isolates were from respiratory sites and only 9.2% were from blood/peritoneum. All of the isolates examined in the current study were from blood culture. In addition, the distribution of isolates from geographic sites was different in the Pennini et al. (2017) study compared to our study. A higher proportion of isolates were from Europe (38% for Pennini et al. versus 19% for our study) and a lower proportion of strains were from Africa (5% for Pennini et al. versus 32% for our study). This may explain the difference in serotype distribution that we observed amongst MDR strains compared to Pennini et al. (2017). Collectively, the data suggests that it is important to prevent *K. pneumoniae* O1 infections due to their high prevalence and virulence and serotypes O2 and O5 due to their association with antibiotic resistance.

A high prevalence of K2 isolates among 519 invasive strains was similarly observed by Paczosa and Mecsas (2016). K24 was the most common capsule type in our study. The hypervirulent K1 capsule type (Siu et al., 2011) was found amongst isolates collected from United States, Taiwan, South Africa, DR Congo, and Pakistan by *wzi* capsular typing. Diverse K types were identified as has been previously reported (Cryz et al., 1986b), indicating that a vaccine that targets only 4 O antigens would be less difficult and less expensive to construct and provide broad coverage compared to a vaccine that targets the capsular polysaccharide (that would need 24 K antigens to provide the same coverage).

Our data showed that clonally diverse *K. pneumoniae* caused bacteremia in patients in 13 countries. Additionally, there was no association between particular O serotypes and sequence types. Therefore, MLST data cannot be used as a proxy for O typing. Furthermore, a strategy to target antigens specific for certain STs might be insufficient to prevent infections caused by diverse lineages of *K. pneumoniae*.

In summary, we determined that there are 4 major O antigen types among 645 strains and 49 K antigens among 519 strains collected from 13 countries. In addition, diverse STs and a high rate of MDR *K. pneumoniae* was determined. Therefore, we believe that a *K. pneumoniae* O antigen-based vaccine would offer an excellent alternative strategy to a vaccine based on capsular polysaccharide which has previously been proposed, developed and evaluated in a Phase 1 study (Edelman et al., 1994). Furthermore, by preventing *K. pneumoniae* infections from occurring in the first place, this will eliminate the need for antibiotics and will therefore remove selective pressure for resistance (Klugman and Black, 2018). Additionally, prevention is far superior to treatment as those patients who survive sepsis suffer multiple long-term complications. Our data suggests that our proposed quadrivalent vaccine (Hegerle et al., 2018) could potentially prevent 90.1% of *K. pneumoniae* bacteremic infections worldwide.

## Data Availability Statement

The original contributions presented in the study are included in the article/Supplementary Material. Further inquiries can be directed to the corresponding author.

## Author Contributions

ST, RS, and AC designed the study. MC, ST, RS, and AC performed the analysis and wrote the manuscript. MT, JKJ, SM, JT, VF, AA, ET, DD, FM, KK, EC, YS, Y-TL, SaS, OP, AS-M, JJ, and OL collected the strains. NH, JN, SuS, SJ, SN, ShS, JP-B, and JS performed the assays. All authors listed have made a substantial, direct and intellectual contribution to the work, and approved it for publication.

## Conflict of Interest

ST, RS, and AC are holders of US patent 9,988,426 titled “Broad spectrum conjugate vaccine to prevent *Klebsiella pneumoniae* and *Pseudomonas aeruginosa* infections.” The remaining authors declare that the research was conducted in the absence of any commercial or financial relationships that could be construed as a potential conflict of interest.

## Footnotes

1 https://mapchart.net/

2 www.pasteur.fr/mlst

3 https://bigsdb.pasteur.fr/klebsiella/klebsiella.html

## Supplementary Material

FIGURE S1 The number of *K. pneumoniae* isolates analyzed by year and country.

FIGURE S2 The number of *K. pneumoniae* isolates tested by each method.

FIGURE S3 Trends in O antigens of invasive *K. pneumoniae* collected from University of Maryland Medical Center in 2011–2015.

FIGURE S4 Distribution of K types by wzi gene sequencing among 519 isolates. Allele# represents wzi alleles which have been assigned an allele number by the wzi database at http://bigsdb.web.pasteur.fr but which do not correspond to a K type.

TABLE S1 Sequence types of 39 isolates with O1, O2, O3, O4, and O5 antigens.

TABLE S2 Resistance of 591 *K. pneumoniae* isolated from blood to 12 different antibiotics.
