# Supplementary material for: The Diversity of Lipopolysaccharide (O) and Capsular Polysaccharide (K) Antigens of Invasive Klebsiella pneumoniae in a Multi-Country Collection
Source: Front Microbiol. 2020 Jun 12;11:1249. doi: 10.3389/fmicb.2020.01249 (PMC7303279; doi:10.3389/fmicb.2020.01249)
Supplement: FIGURE S1 — The number of K. pneumoniae isolates analyzed by year and country. [file Data_Sheet_1.PDF]

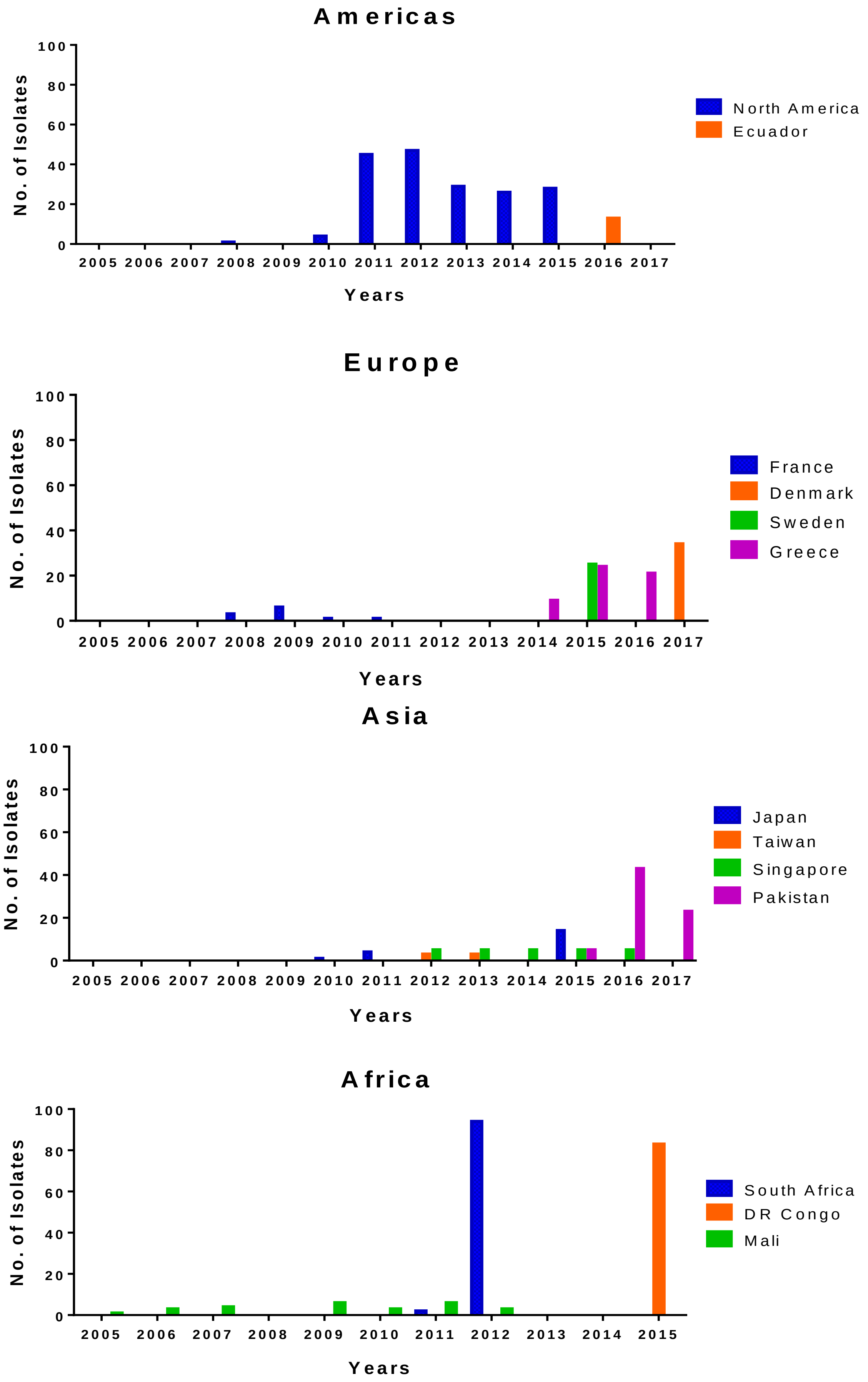

Figure S1. The number of *K. pneumoniae* isolates analyzed by year and country.

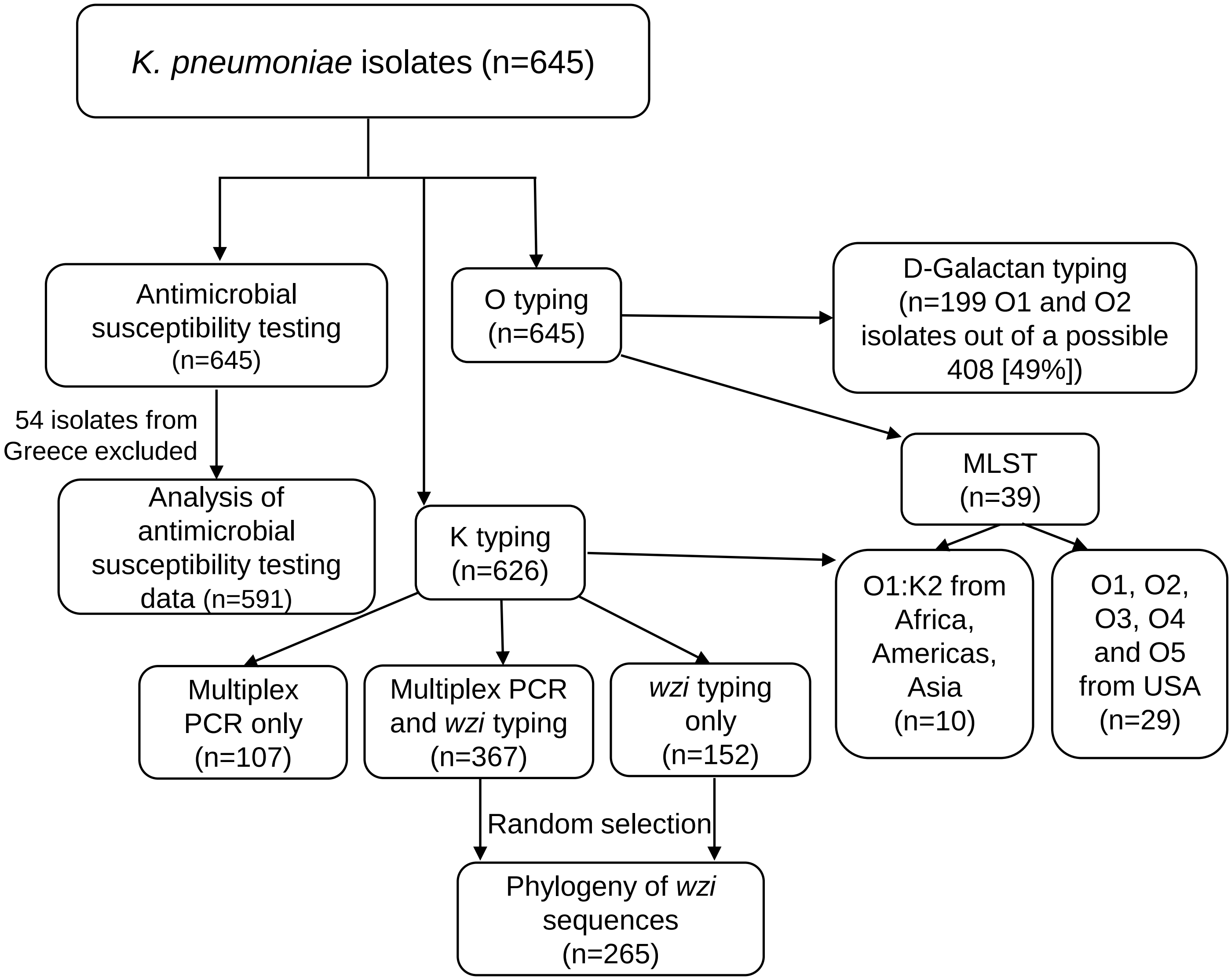

Figure S2. The number of *K. pneumoniae* isolates tested by each method.

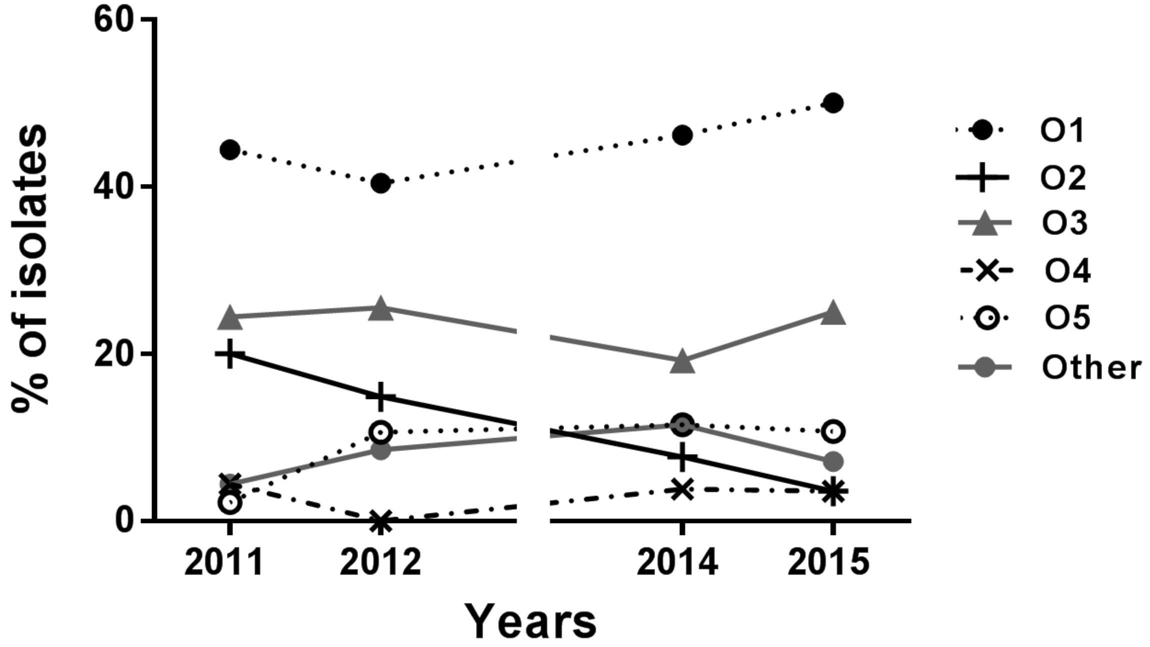

Figure S3. Trends in O antigens of invasive *K. pneumoniae* collected from University of Maryland Medical Center in 2011-2015.

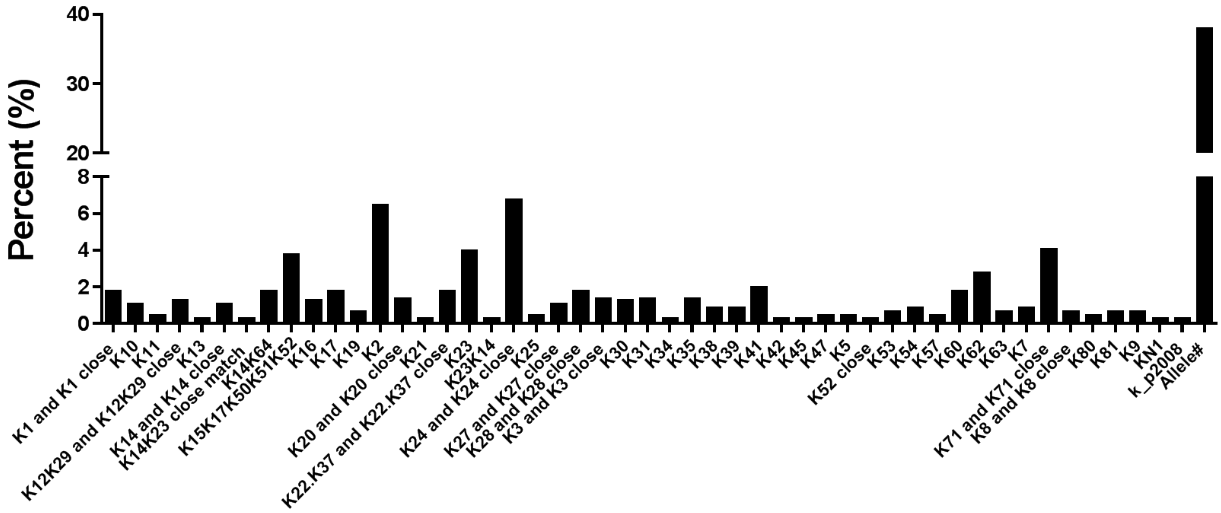

Figure S4. Distribution of K types by *wzi* gene sequencing among 519 isolates. Allele # represents *wzi* alleles which have been assigned an allele number by the *wzi* database at http://bigsdb.web.pasteur.fr but which do not correspond to a K type.
